# Union of Fractured Bones

**Published:** 1842-03

**Authors:** 


*Union of Fractured Bones.*—The following cases which we extract from a paper published by A. Thierry, in L'Experience, Nov. 4, 1841, are curious and also practically important, as illustrating the dependence of the reparative process on the state of general health. The fact that the existence of a venereal taint in the system will so seriously interfere with the formation of a callus, and the consolidation of the fractured bone, is new to us.

*Case* 1. M. aged 13, clerk in a wine store, fractured both bones of the fore-arm in descending into the vaults. The apparatus of Boyer was applied, and at the end of sixty days removed, the union being supposed to be perfect. On examination the consolidation was not complete, yet this young man enjoyed good health, and there was nothing to explain why the fracture did not unite. The patient was closely watched and it was soon discovered that the girl who nursed him, afforded him from time to time a physical solace for his ennui; she was discharged, the apparatus re-applied for sixty days and the consolidation was complete.

*Case* 2. A school boy of 14 had the arm broken by the fall of a bench. The displacement was inconsiderable, the fracture was reduced and put in the old apparatus; on removing the dresssings, the bone had not united. The boy was watched and it was discovered that he was addicted to masturbation. The apparatus was re-applied and means taken to prevent his recurring to his old habit. The cure was complete.

*Case* 3. M. D. making a false step in the street, fell and fractured the thigh above the great trochanter. The fracture was reduced and the apparatus of Boyer applied. At the end of sixty days, on removing the apparatus, the bone had not united. The patient was asked whether he had not some venereal affection; on his replying in the affirmative an anti-venereal course was pursued, the apparatus was re-applied, and in twenty four days the fracture was consolidated.—*N. Y. Med. Gaz.*

